# Transfemoral amputation and prosthesis provision in Tanzania: Patient and provider perspectives

**DOI:** 10.4102/ajod.v12i0.1084

**Published:** 2023-02-14

**Authors:** Mayur Urva, Claire A. Donnelley, Sravya T. Challa, Billy T. Haonga, Saam Morshed, David W. Shearer, Nooshin Razani

**Affiliations:** 1 Department of Orthopedic Surgery, Institute for Global Orthopaedics and Traumatology, University of California, San Francisco, United States; 2 Department of Orthopaedics and Rehabilitation, Yale School of Medicine, New Haven, United States; 3 Department of Orthopaedic Surgery, Harvard University, Boston, United States; 4 Department of Orthopaedic Surgery, Muhimbili Orthopaedic Institute, Dar es Salaam, United Republic of Tanzania; 5 Orthopaedic Surgery, Institute for Global Orthopaedics and Traumatology, University of California, San Francisco, United States; 6 Department of Epidemiology and Biostatistics, University of California, San Francisco, United States

**Keywords:** transfemoral amputation, low-resource setting, prosthetic rehabilitation, barriers, qualitative, thematic analysis, caregiver, Tanzania

## Abstract

**Background:**

The burden of disability because of traumatic limb amputation, particularly transfemoral amputation (TFA) is disproportionately carried by low- and middle-income countries. The need for improved access to prosthesis services in these settings is well-documented, but perspectives on the burden imposed by TFA and the challenges associated with subsequent prosthesis provision vary among patients, caregivers and healthcare providers.

**Objectives:**

To examine the burden of TFA and barriers to prosthesis provision as perceived by patient, caregiver and healthcare professional, at a single tertiary referral hospital in Tanzania.

**Method:**

Data were collected from five patients with TFA and four caregivers recruited via convenience sampling, in addition to 11 purposively sampled healthcare providers. All participants participated in in-depth interviews regarding their perceptions of amputation, prostheses and underlying barriers to improving care for persons with TFA in Tanzania. A coding schema and thematic framework were established from interviews using inductive thematic analysis.

**Results:**

All participants noted financial and psychosocial burdens of amputation, and perceived prostheses as an opportunity for return to normality and independence. Patients worried about prosthesis longevity. Healthcare providers noted significant obstacles to prosthesis provision, including infrastructural and environmental barriers, limited access to prosthetic services, mismatched patient expectations and inadequate coordination of care.

**Conclusion:**

This qualitative analysis identifies factors influencing prosthesis-related care for patients with TFA in Tanzania which are lacking in the literature. Persons with TFA and their caregivers experience numerous hardships exacerbated by limited financial, social and institutional support.

**Contribution:**

This qualitative analysis informs future directions for research into improving prosthesis-related care for patients with TFA in Tanzania.

## Introduction

As of 2017, an estimated 57.7 million people were living with traumatic limb amputations (McDonald et al. 2021) with this disability burden disproportionately carried by low- and middle-income countries (LMICs) (Harkins, McGarry & Buis 2013). While in high-income countries (HICs) amputation is typically a complication of peripheral vascular disease or diabetes, trauma as a cause of amputation is more common in LMICs such as Tanzania (Agu & Ojiaku 2016; Chalya et al. 2012; Gebreslassie, Gebreselassie & Esayas 2018; Grudziak et al. 2017; Ogeng’o, Obimbo & King’ori 2009; Thanni & Tade 2007). Among amputations, it is reported that the incidence of transfemoral amputations (TFAs) is up to 34% (Grudziak et al. 2019). Patients sustaining a traumatic TFA have a 68% increase in metabolic cost, or the fatigue caused by ambulation and the impetus for the development of above-knee prostheses has been to restore mobility and decrease this metabolic cost (Czerniecki 1996). Prostheses also offer persons with TFA increased opportunities to participate in society and be economically productive (Donnelley et al. 2022; Harkins et al. 2013), but the large unmet need in low-resource settings extends beyond just the costs of prostheses (Lemaire, Supan & Ortiz 2018).

Low- and middle-income countries often cannot prioritise prosthesis services because of strained resources, no access to prosthesis materials, and a lack of established models of prosthesis provision, leading to an estimated 29m individuals in resource-limited environments that are in need of orthotic and prosthetic services (Harkins et al. 2013; Jesus & Hoenig 2019). A single trained prosthetist can treat about 250 people annually, suggesting that based on estimates, Eastern Sub-Saharan Africa requires 2400 prosthetists (World Health Organization [WHO] 2005) and associated centres equipped to deliver prosthesis care. However, despite the high disability burden, prosthetics and orthotics training programmes exist in less than 25% of developing countries (WHO 2005). Institutions such as the Tanzania Training Centre for Orthopaedic Technologists (TATCOT), the primary prosthetics and orthotics training institute in East Africa, have attempted to address this gap, but as of 2012, only about 425 prosthetists had been fully trained (Sexton, Shangali & Munissi 2013). In addition, prostheses from industrialised countries are often not well designed for the specific environmental and lifestyle needs of low-resource settings, and the technology can require regular maintenance that makes the sustainability of these interventions challenging (Donnelley et al. 2021; Harkins et al. 2013; Meanley 1995).

The challenges faced by persons with amputations, particularly those with lower-limb amputations, are well-documented (Andregård & Magnusson 2017; Shaw et al. 2018), but perspectives differ among patients, caregivers and healthcare providers (Allen et al. 2020; Ibrahim et al. 2019; Song et al. 2019), especially regarding the role of prosthesis provision and obstacles to providing appropriate care. The purpose of this study, therefore, was to examine: (1) the burden of TFA experienced by patients and caregivers, and (2) the challenges associated with prosthesis provision as experienced by patient, caregiver and healthcare professional, at a single urban community in Tanzania.

## Research methods and design

### Study design

This qualitative study involved 20 semi-structured interviews of patients with TFA, their caregivers, and healthcare providers from the orthopaedic surgery, orthotics and prostheses, and social work departments of Muhimbili Orthopaedic Institute (MOI). Muhimbili Orthopaedic Institute is a tertiary referral hospital in Dar es Salaam, Tanzania, with a high volume of orthopaedic trauma. Muhimbili Orthopaedic Institute has one of only a few prosthesis fabrication workshops in the county.

### Recruitment

**FIGURE 1:**
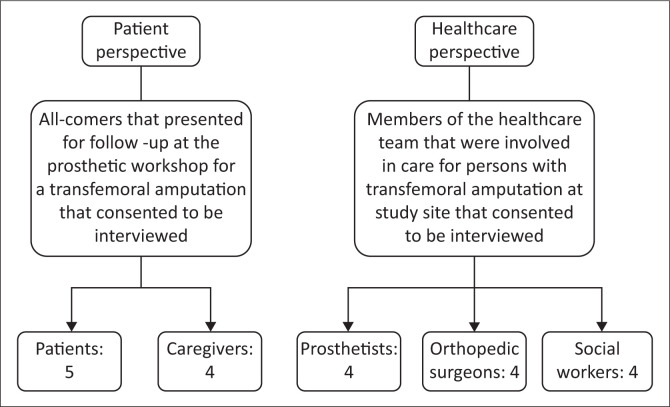
Participant recruitment.

The study identified patients from a concurrent, larger prospective cohort study of 30 patients with TFA provided with definitive modular endoskeletal transfemoral prostheses; the design and methods of this study have been previously reported (Von Kaeppler et al. 2021). Patients and caregivers presenting to MOI Prosthetics and Orthotics workshop for follow-up of the prospective cohort study were screened for eligibility from November 2017 to January 2018, and were eligible if the participant was over 18 years of age, if patient completed a unilateral TFA between 6 months and 2 years prior to recruitment and if willing to provide recorded verbal consent. Participants were not eligible if the patient currently uses or previously used a prosthetic limb at the time of recruitment, or were unable to verbally comprehend and respond to interview questions. Purposive sampling was used to identify groups of healthcare providers practising at MOI with different expertise (prosthetists, orthopaedic surgeons and social workers) to incorporate perspectives from multiple steps of prosthesis provision, including amputation, prosthesis fitting and rehabilitation. The study was announced to all providers from the above departments and volunteers were recruited. Recruitment ended when data saturation was reached and additional interviews were yielding diminishing returns of new information. A summary of the participant recruitment can be seen in Figure 1.

### Ethical considerations

The study was approved by the National Institute for Medical Research (NIMR) in Tanzania, as well as the Institutional Review Board (IRB) at University of California, San Francisco (IRB # 19-27566). The Institutional Review Board issued an ethics exemption for the study because it is research that only includes interactions involving educational tests, survey procedures, interview procedures or observation of public behaviour. Participants were informed about the study purpose and reassured that their responses would not affect their participation in the concurrent prospective cohort study. Recorded verbal consent was provided at the beginning of the interview.

### Interviews

Interviews were conducted at MOI between November 2017 and January 2018. Interviews were conducted in English or Swahili per interviewee preference, by a study investigator with experience in qualitative study design. Where needed, Swahili translation was provided by one of three trained local research coordinators. Field notes were recorded during and after interviews, which typically lasted 45 min – 1 h.

All interviews addressed: (1) the impact of TFA in an urban Tanzanian community and (2) the process of prosthesis provision. A template of questions was designed by the study authors based on literature review, with input from prosthetists, orthopaedic surgeons, and social workers at the home and partner institutions. All interviews began with a standardised, open-ended questions, followed by questions tailored to participant background; a list of potential participant-specific questions that was used as an interview guide can be seen in Table 1- A1. Interviewees were then prompted to address the following topics: financial challenges, changes to social relationships and role in the community, and challenges with receiving and providing medical and prosthetic care related to the TFA. In the second stage of the interview, patients and caregivers were asked what changes they expect after receiving their prosthesis, while healthcare professionals were asked what challenges they have experienced or witnessed while providing prostheses.

### Data analysis

Audio recordings were transcribed verbatim in Microsoft Word and imported into Atlas.ti qualitative analysis software (Scientific Software Development GmbH) (Thomas 2006). Utilising a subset of transcripts, two investigators (M.U, C.A.D) developed an initial coding framework inductively based on emerging themes and ideas. Code groups were defined as repeated themes, and codes were defined as categories within the code groups. A comprehensive list of codes and code groups was established, and all investigators reviewed the coding scheme. Atlas.ti (Mac versions 8 and 9) was used to code the interviews independently and in duplicate by investigators (M.U, C.A.D) according to the coding schema (Table 1 - A2). Informal intercoder reliability assessments were conducted throughout the coding process to ensure consistency in the utilisation of codes between investigators and establish trustworthiness of data analyses (Lincoln & Guba 1985).

Next, two investigators (M.U, C.A.D) employed inductive thematic analysis (Thomas 2006), whereby themes were developed from the coded text and incorporated into a conceptual framework (Table 1). The research team used this framework to identify perceived connections between: (1) burden of amputation on patients and caregivers, (2) expectations of and perceived impact of prostheses and (3) obstacles to providing prostheses to persons with TFA. Anonymised quotations were extracted to support the thematic analysis and to highlight the perspectives of the different stakeholders who were interviewed. A collection of supporting quotations is included as Table 1 - A3.

**TABLE 1:** Thematic overview by participant group.

	Patient and/or caregiver	Healthcare professional
Perceived impact of amputation	- Financial impact	- Financial impact
	- Psychosocial impact	- Social impact
	- Caregiver burden	
Perceived impact of prostheses	- Return to normality	- Return to normality
	- Ability to work and provide for family	- Good longevity
Perceived obstacles to prosthesis provision	- Difficulty accessing and affording prosthetic care	- Difficulty accessing and affording prosthetic care
	- Limited knowledge of rehabilitation and prostheses	- Burdensome rehabilitative process
	- Prosthesis longevity	- Poorly adapted to Tanzanian environment
	- Limited governmental support	- Mismatched patient expectations
		- Poor coordination of care between surgeons and prosthetists
		- Limited governmental awareness and support

## Results

### Participants

The final study population included 20 individuals: (1) 5 patients with TFA, (2) 4 ‘caregivers’ defined as individuals responsible for a large component of the care of the person with a TFA, and (3) 11 healthcare providers representing 3 professions, namely orthopaedic surgeons (*n* = 4), prosthetists (*n* = 4) and social workers (*n* = 3). Patients were predominantly male (87%), and of average age of 18 years; subset of data from concurrent prospective cohort study (Weir, Ephraim & Mackenzie 2010). Of the five patients with TFA, one had received a prosthesis by the time of the interview, and the remaining patients were scheduled to receive prostheses through the concurrent prospective cohort study.

### Patient and caregiver interviews

#### Perceived impact of amputation

Patients and caregivers identified financial and psychosocial consequences of TFA. Many persons with TFA are of working age in vocations requiring manual labour, and amputation led to the loss of employment. Social stigma and loss of agency contributed to increased feelings of hopelessness and isolation:

‘Before amputation, I was just normal. I was able to go anywhere I want and do anything I want to do. Since the amputation, I don’t do anything. I just stay at home. It isn’t okay with me, but I just have to accept the fact that it is done.’ (Record 49, Patient, 20 December 2017)

‘I have lost a lot of friends and been rejected to the bigger community, which annulled me because they thought I’m a beggar. If I tried to call to say hi, they thought I would ask for help. So I lost a lot of friends and some relatives as well. […] Most of my friends never answer my phone calls and I have very few people who are still with me.’ (Record 30, Patient, 09 December 2017)

Caregivers in particular reported that the increased responsibility of caring for a person with TFA often caused a decrease in their own ability to provide for their family, due to the burden of time and responsibility towards the person with TFA:

‘I have spent all of my money and resources for my father’s treatment. When I was a taxi driver, I used to make money. But, since my father’s amputation, I haven’t had a proper income to support my family, my father, or myself.’ (Record 99, Caregiver, 23 December 2017)

#### Perceived impact of prosthesis

Patients and caregivers were optimistic about the potential impact of receiving a prosthesis. Participants hoped for increased independence and re-establishing their participation in the workforce and communities:

‘I think [*having a prosthesis*] will change my life 100%, because I will finally be able to walk again, do my own domestic activities as well as my small business as I used to. I can also interact with my community in a way that I want to.’ (Record 5, Patient, 12 January 2018)

#### Perceived obstacles to prosthesis provision

Patients had limited knowledge of the rehabilitative process and how to access prosthetic care:

‘We came for two visits to see the surgeon after [patient’s] amputation, when we were advised about PT [*physical therapy*] but we weren’t told about prosthetics [*until later*]. It seems like knowledge of where and how we can get a prosthetic has been lacking, and that’s the biggest hurdle to his care so far.’ (Record 99, Caregiver, 23 December 2017)

A widely perceived limitation of prostheses was prosthesis management after initial provision. The cost of prosthesis repair and replacement dampened expectations for ongoing independence and for prosthesis longevity:

‘Once we get the prosthetic and the follow-up is finished […] we will try our best to save up to get a new prosthetic. I don’t know how long this prosthetic will last.’ (Record 50, Caregiver, 21 December 2017)

### Healthcare professional interviews

#### Perceived impact of amputation

Healthcare professionals recognised the significant financial and social burdens of TFA:

‘For most [patients], they are the breadwinners of their family, and they are responsible for taking care of their families. So after getting an amputation, they are not able to care for their families […] To them, amputation is the end of their lives as they know it.’ (Record 2, Social Worker, 05 January 2018)

#### Perceived impact of prosthesis

Healthcare professionals saw prostheses as a chance for patients to return to previous activities:

‘We can see even after providing a prosthetic, they still don’t have their own limb but they are able to return to functioning and working.’ (Record 20, Prosthetist, 28 December 2017)

#### Perceived obstacles to prosthesis provision

Healthcare workers noted several limitations to prostheses provision in Tanzania, predominantly the lack of access. Government awareness, in addition to minimal support and unavailability of prosthetic services was noted, particularly in rural regions:

‘… there are two [*Prosthetic and Orthotic Centers*] in Dar [*es Salaam*]. There are about 60 million people and less than 10 centers in the country.’ (Record 3, Prosthetist, 02 January 2018)

‘Out of 100 people at the Ministry of Health, maybe two or three are aware of our department and what we do. Even when they see people with prosthetics, they likely think that the patients were treated internationally.’ (Record 20, Prosthetist, 28 December 2017)

Patients that have a nearby prosthetist centre and can receive a prosthesis still need to navigate a long and difficult rehabilitative process:

‘We have very few centers, so we have patients traveling from far away to Dar es Salaam […] we need time to make a prosthetic, from two weeks to 1 month […] if it’s one month, these patients coming from elsewhere have to stay here, which costs a lot of money […] so things like gait training get shortened, because the patient has to go back home before they are able to walk well on the prosthetic.’ (Record 4, Prosthetist, 09 January 2018)

Prosthetists observed that prosthesis longevity can be significant despite the challenges of maintenance and repair. However, replacement can be costly and difficult because of resource limitations:

‘Since we have been providing prosthetics for quite a long time, we can see that amputees can use prosthetics for even 10 years. We can replace the socket or foot for free when the patient needs it.’ (Record 1, Prosthetist, 27 December 2017)

‘The problem will happen when these knee joints get broken. We might not have other joints to replace these broken joints, which means that they might have to go to a different joint and learn about that, get used to that.’ (Record 4, Prosthetist, 09 January 2018)

Poor functionality and adaptive capability for the current infrastructure in Tanzania were noted to further limit the potential impact of prostheses:

‘Apart from poverty, the environment is a barrier to prosthetic care. For example, sometimes the prosthesis we make for patients can’t be used on muddy ground, but that’s very difficult in Tanzania. For instance, today it has been raining and most of our patients don’t have private cars. There is no way to escape the mud. The infrastructure in this country is not suitable for prosthetics.’ (Record 3, Prosthetist, 02 January 2018)

Inadequate counselling about these limitations can cause a mismatch between patient expectations and reality, which in turn can lead to disappointment and prosthesis abandonment:

‘The other day, I did a prosthetic […] the patient didn’t like the cosmetic cover because it didn’t match the color of his skin […] His prosthetic was fit to be used by a farmer, as he needs it to fit in gumboots, etc. At the end of the day, he left the prosthetic because he didn’t understand how much it could impact his life. […]’. (Record 20, Prosthetist, 28 December 2017)

Operative planning in the setting of TFA was largely determined by surgeon discretion without input from prosthetists, which could limit stump-site suitability for prosthesis:

‘We don’t normally consider how much [of the stump] to retain for future rehabilitation, because prosthetics are really expensive and most of our patients cannot afford them […] Now, surgeons operate and leave prosthetics and orthotics to deal with the stump.’ (Record 100, Surgeon, 27 December 2017)

## Discussion

To the authors’ knowledge, this study is the first to qualitatively compare patient, caregiver and healthcare perspectives of TFA in Tanzania. Such findings contextualise infrastructural limitations and further describe local perceptions of TFA and prosthesis provision, informing strategies to improve care for patients with TFA in low-resource settings.

The caregiver burden in the setting of chronic disability in HICs is well documented (Altieri & Santangelo 2021; Tsoulou et al. 2019; Weir et al. 2010), but few studies have examined the caregiver burden in LMICs, despite the higher disability burden (Gajraj-Singh 2011; Leurs et al. 2018; Schulz, Visintainer & Williamson 1990; Thrush & Hyder 2014). The findings of this study demonstrate that the psychosocial and economic burden of TFA extends beyond the patient. While all participants noted the impact of TFA on patients, caregivers also experienced isolation, loss of employment and hopelessness in the aftermath of their family member’s amputation. Particularly in the case of elderly patients whose adult children take on the role of caregiver, the burden of caring for the person with TFA depreciated the caregiver’s ability to fulfil their other roles as a parent and family member.

The impact of amputation quality on rehabilitative outcomes is well described (Cosgrove et al. 2002; Sooriakumaran et al. 2018). Healthcare providers from all professions noted that the lack of care coordination between specialties, or the lack of surgical awareness that prosthesis provision was possible, led to surgical planning that did not include stump-site preparation, making eventual rehabilitation and prosthesis provision more challenging if the residual limb was not suitable for a future prosthesis. This is supported by the work of Kam et al. (2015) who found that inappropriate surgical procedures that affected prosthesis fit and a lack of follow-up care were major barriers to rehabilitation of persons with amputations in low-income countries. One solution implemented at the study site after interviews were completed was incorporating a prosthetics and orthotics elective into the orthopaedic residency curriculum, which improved the surgeons’ understanding of planning for prosthesis provision.

One prominent theme in this study was mismatch between patient expectations and reality, which may lead to prosthesis abandonment. Prosthesis abandonment has been previously reported in this population as high as 30% (Von Kaeppler et al. 2021). To patients and caregivers, the idea of a prosthesis represents a complete return to life before amputation, whereas prosthetists may have a more realistic understanding of the ongoing rehabilitation and unique challenges to long-term prosthesis use. For example, prostheses in low-resource settings require specific functionality to allow for navigating uneven terrain and environmental conditions, in addition to participating in cultural norms such as squatting and genuflecting to elders (Kam et al. 2015; Marino et al. 2015; Meanley 1995). Prostheses with such capabilities are likely not accessible, while lower-cost prostheses with less ability to replicate native knee kinematics may be available. Prosthetists also noted how cosmesis changed whether patients felt comfortable using their prosthesis in the long term. Properly managing expectations with perioperative and postoperative counselling and utilising prostheses specifically designed for the Tanzanian environment and cultural context may help with ensuring their long-term impact.

Based on the findings of this study, long-term impact of prostheses is limited by prosthesis longevity and institutional support, a fact recognised by patients, caregivers and healthcare providers alike. The cost of repair and replacement is a primary cause of prosthesis abandonment, and external funding is often necessary, but insufficient, to care for the total population of persons with amputations (Harkins et al. 2013; Marino et al. 2015).

Therefore, further exploration of government-driven interventions is essential. Ennion et al. reported some challenges of implementing disability grants for persons with amputation in rural South African communities, where they provided essential temporary support but did not always improve adherence to a difficult and frustrating rehabilitation process (Ennion & Johannesson 2018). In the current study, patients often expressed a desire for increased support in the form of employment protections and entrepreneurial grants. Healthcare providers hoped that manufacturing and import costs and gaps in health insurance coverage could be addressed in the future. To ensure true sustainability, country-specific interventions will need to consider various environmental, socioeconomic, and cultural nuances, and incorporate stakeholders from multiple perspectives.

This study had several limitations. Only one patient had already received a prosthesis at the time of interview and prostheses were provided free of charge. Therefore, patient and caregiver perspectives of prosthesis benefits and challenges were largely speculative and possibly more positive than if they were not provided for free. Furthermore, the dominance of the provider view led the results to tend towards service provision rather than the experience of limited access.

Additionally, while this study contributes a Tanzanian perspective to the literature, it only includes a single site at a specialised urban centre. Including rural Tanzanian communities would have provided a more comprehensive perspective. Finally, capturing more information about occupation, age, gender and family role would have allowed for a deeper exploration of the interaction of these domains with the themes that emerged from the study analysis. Despite these limitations, this study sought to include the perspective of several relevant stakeholders, including patients, caregivers and healthcare workers from different professions. The study also explored factors such as the impact of poor multidisciplinary communication and mismatched patient expectations, that have been less widely reported.

## Conclusion

Transfemoral amputation in Tanzania carries a significant financial, psychological and social burden for patients and caregivers alike. The quality of prosthesis provision is hampered by poor suitability to the current infrastructure, financial limitations to prosthetic services, mismatched patient expectations and healthcare management. These data support the need for further study into prosthesis longevity in LMICs and methods to improve access to rehabilitation as well as financial and psychosocial support for amputees and their caregivers.
